# Acceptability of Male Circumcision for the Prevention of HIV/AIDS in the Dominican Republic

**DOI:** 10.1371/journal.pone.0007687

**Published:** 2009-11-02

**Authors:** Maximo O. Brito, Lilliam M. Caso, Hannabell Balbuena, Robert C. Bailey

**Affiliations:** 1 From the Section of Infectious Diseases and the School of Public Health, University of Illinois at Chicago, Chicago, Illinois, United States of America; 2 From Pasantía Rural, Secretaría de Salud Pública, Santo Domingo, Dominican Republic; U.S. Naval Medical Center Research Detachment, Peru

## Abstract

**Background:**

Male circumcision (MC) is an effective strategy to prevent HIV infection in heterosexual men. To our knowledge, there are no studies of the acceptability of this procedure in the Dominican Republic (DR). The main objective of this study was to assess the acceptability of MC to prevent HIV transmission among men ages 18 to 50 years in the Altagracia Province in the Dominican Republic. Because differences in culture and beliefs between Haitians and Dominicans could potentially influence their acceptability of MC, we conducted a comparative analysis based on national origin.

**Methods:**

A survey was administered to a convenience sample of 368 men. The questionnaire was divided in 3 sections: 1) Background demographics (including national origin), 2) Male circumcision and 3) Sexual health. Stratified and logistic multivariate regression analyses were performed to identify factors associated with the acceptability of MC.

**Results:**

The sample consisted of 238 (65%) Dominicans and 130 (35%) Haitian immigrants. Almost all participants were uncircumcised (95%) and about half (52%) were single. The overall acceptability of MC was 29%. The number of men willing to be circumcised increased to 67% after an information session explaining the benefits of the procedure. 74% of men reported that they would be willing to circumcise their sons after hearing that information. In multivariate analysis, Haitian nationality (OR = 1.86, 95% CI 1.01–3.41), knowing that circumcision improves hygiene (OR = 2.78, 95% CI 1.29–6.0) and not believing that circumcision decreases sexual pleasure (OR = 2.18, 95% CI 1.20–3.94) were associated with a higher acceptability of the procedure. Although age was not significantly associated with the willingness to be circumcised in the multivariate analysis, stratified analysis based on national origin suggested that younger Dominicans (<30 years of age) are more likely to accept the procedure when compared to their older counterparts (OR = 2.17, 95% CI 1.14–4.12).

**Conclusions:**

An important number of sexually active men in the DR may be willing to be circumcised if educational resources detailing the benefits of the procedure are made available. These educational activities would constitute a great opportunity to teach about sexual health and reinforce safe sex practices.

## Introduction

Three randomized clinical trials have shown that male circumcision (MC) reduces the risk of HIV infection by 50–60% in heterosexual men [1]–[3]. These findings are likely to increase the demand for safe and affordable MC services in areas of moderate to high HIV prevalence where the procedure is not routinely performed. A recent survey found that no single country in Central or South America has a MC prevalence of greater than 20% [4]. In the Dominican Republic (DR), a 2007 nationwide Demographic and Health Survey (DHS) found that approximately 86% of men between the ages of 15–59 were uncircumcised [5]. The same survey estimated that 94% of men in the Altagracia province were uncircumcised.

AIDS is the leading cause of death in the Caribbean basin among people aged 15–44 years. There are approximately 230,000 people living with HIV in the region, the majority residing in the DR and Haiti [6]. The prevalence of HIV infection in the DR is approximately 0.8% nationwide and 1.2% in the Altagracia Province [5]. This province has a significant number of “bateyes”, the communities surrounding sugar cane plantations, where the prevalence of HIV is higher than in the general population (3.2%) [7]. A mixed population of Dominicans and Haitian migrant workers inhabits these communities. Heterosexual intercourse is typically regarded as the principal mode of HIV transmission in the DR and the rest of the Caribbean countries [6], however, some authors have recently proposed that “bisexuality” may be an important factor driving the DR epidemic [8].

The acceptability of male circumcision has been studied in men and women in several countries in Sub-Saharan Africa [9], in men who have sex with men (MSM) in China [10] and the United States [11], in Indian mothers [12] and in expectant parents and men in Thailand [13]–[15]. These studies show a wide variation in the willingness to circumcise ranging from 14.2% in Thailand [15] to more than 80% in Swaziland [16]. To our knowledge, only one acceptability study has been conducted in Latin America [17]. This survey based study of 2048 MSM in Ecuador and Peru, found an overall circumcision prevalence of 3.7% and an acceptability rate of 54.3% in these countries.

The primary aim of the present study was to evaluate the acceptability of male circumcision to prevent HIV infection among sexually active men in the Altagracia Province, an area of moderate HIV prevalence and low circumcision rates. Because differences in culture and beliefs between Haitians and Dominicans could potentially influence their acceptability of MC, we felt it was important to compare these two groups.

## Methods

### Ethics statement

*All participants were informed of the risks and benefits of the study and were provided with a “Subject Information Sheet” detailing the study. Consent was obtained. A waiver of consent documentation was granted by the Institutional Review Board of the University of Illinois given that the research met criteria for “minimal risk” to the subjects. The subject's name was not recorded and all data was de-indentified. Subjects did not receive compensation for participating in this study. The entire research protocol was approved by the Institutional Review Board at the University of Illinois and by the independent research review board of the Dominican Foundation of Infectious Diseases.*

*A letter of support was obtained from authorities of the Ministry of Health of the Altagracia Province*.

The study was conducted in July –August 2008 in 19 communities of La Altagracia Province in the Dominican Republic. The communities surveyed were selected from the five municipalities that make up the province in an attempt to obtain representation from a broad geographic area. The subjects were recruited randomly from parks, bus stops, neighborhoods (door to door) and “motorcycle taxi” stops. Members of the research team approached every other man they encountered at these locales. Individuals within the target age range for the study (18–50 years) were invited to participate.

A convenience sample of 300 individuals divided by age groups (“younger” = 18–30 year-olds and “older” = 31–50 year-olds) and national origin (Dominican and Haitians) was planned at the beginning of the study. The intent was to recruit 100 Dominicans and 50 Haitians per age category. Approximately half of the participants were to be recruited from rural communities. The number of participants and their nationalities were recorded in an Excel spreadsheet at the end of each working day. Recruitment of the 300 participants was completed earlier than expected but the investigators decided to continue the interviews and increase the sample size.

The inclusion criteria for participating in the study were:

1. Men between the ages of 18 and 50.
2. Being able to speak and understand Spanish or English.

The exclusion criteria were:

1. Individuals with hearing or speech impairment.
2. Children less than 18 years of age and adults older than 50 years.

All study materials were translated from English to Spanish by the investigators.

A survey was designed with the primary aim of obtaining information on the subject's acceptability of male circumcision as an effective strategy to prevent HIV infection (“willingness to be circumcised”). Other demographic, knowledge and behavioral variables were included to ascertain their influence in the participant's willingness to be circumcised. The survey consisted of 25 questions divided in 3 sections:

1) Background demographics, 2) Male circumcision and 3) Sexual health. In the first section, participants were asked about basic demographic information such as age, educational level, national origin, religion, marital status and number of children. The second section was designed to determine the subject's circumcision status and their knowledge about the benefits of the procedure. In section three, the questions dealt with sexual health and behaviors.

At the end of the interview, uncircumcised participants were asked if they would be willing to have a circumcision if the procedure was offered. After recording their response, we proceeded to read a statement describing the benefits of MC in decreasing HIV risk and posed the question again. Lastly, we asked if they would be willing to circumcise their children.

### Statistical analysis

Data from the questionnaires was entered in Microsoft Access 2003 and analyzed using SAS version 9.1.3 service pack 4, XP_PRO platform (SAS institute Inc). Descriptive statistics and univariate analysis were generated for each of the variables corresponding to specific questions in the survey.

Bivariate analysis was performed to study the association of each of the independent variables with the main outcome of interest (willingness to be circumcised before an information session). The majority of independent variables were dichotomized based on the presence (“Yes”) or absence (“No”) of the specific characteristic being examined. The variable age was arbitrarily dichotomized as “older” and “younger” using the approximate median age (30 years) of the cohort as the cutoff. The dichotomous variable “marital status” included those who cohabitated with their partners (“Yes”) and the truly single (“No”). Eighth grade was used as the cutoff for the dichotomous variable “education”. Contingency tables were constructed for all of the comparisons and Chi Square (**X^2^**), Mantel-Haenszel statistics and measures of association were calculated (Odd Ratios). Test statistics were considered significant if p values were less than 0.05, and 95% confidence intervals were calculated for the measures of association.

A comparative analysis between Haitians and Dominicans was performed. Contingency tables were constructed using Nationality as the dependent variable and the demographic characteristics, sexual behaviors and rates of self reported STI as the explanatory variables. Chi square statistics were calculated for these associations.

Prior to modeling, a stratified analysis was done to determine if nationality (Haitian vs. Dominican) was a confounder or an effect modifier of the relationship between age and willingness to circumcise. We also looked at nationality and willingness to circumcise stratified by age (younger vs. older). These analyses were done to determine if the effect of the variables was independent of each other, given their significant association with the outcome in the bivariate analysis. The data on willingness to circumcise before and after the information session were analyzed separately.

Multivariate logistic regression analysis was performed to identify factors associated with the acceptability of MC before the information session. Included in the model were variables that showed a statistically significant (p = <0.05) association with the willingness to be circumcised in the unadjusted analysis. Although educational level did not reach statistical significance in the bivariate analysis, it differed between nationalities and hence we felt it was an important variable to include. The final model included the dependent variable “willingness to undergo a circumcision” (before the information session) and the explanatory variables nationality, age (18–30 years/30–50 years), education (<8^th^ grade/>8^th^ grade) and the group of variables that dealt with the perceived benefits of the procedure. Interactions were examined and none were found. Multivariate analyses using the variables of “willingness to circumcise” after the information session were not performed since we did not ask the knowledge or hygiene questions again after reading the statement.

## Results

368 men were interviewed. Fewer than 5% of individuals who were approached and met the inclusion criteria declined to participate in the study. Another 3% of eligible individuals were excluded because did not understand Spanish or English.

Tables 1 and 2 provide an overall description of the study population by national origin. The sample consisted of 238 (65%) Dominicans and 130 (35%) Haitians. Almost all participants were uncircumcised (95%) and about half (52%) were single. The median age was 30 years for Dominicans and 27 years for Haitians (range 18–50).The first sexual encounter occurred at a median age of 14 (range 7–25) for Dominicans and 15 (7–26) for Haitians. The median number of children was 1 (range 0–9) for both groups. Dominicans reported more sexual partners during the previous year (p = 0.01), had a higher level of formal education (p = 0.01) and were more likely to have been tested for HIV in the past (p = <0.01).

**Table 1:** Subject characteristics by national origin.

Category	Subcategory	Dominican	Haitian	Total	χ^2^ (p value)
		n = 238 (65)	n = 130 (35)	368 (100)	
**Median age (18–50 years)**		30	27	29	
**Median age first sex**		14 (7–25)	15 (7–26)	14 (7–26)	
**Median # of children**		1 (0–9)	1 (0–8)	1	
**Religion**	**Catholic**	97 (41)	53 (41)	150 (41)	0.95
	**Non-Catholic**	141 (59)	76 (59)	217 (59)	
**Education**	**<8th grade**	111 (49)	74 (64)	185 (54)	**0.01**
	**>8th grade**	115 (51)	42 (36)	157 (46)	
**Marital status**	**Married**	115 (48)	60 (46)	175 (48)	0.69
	**Single**	123 (52)	70 (54)	193 (52)	
**Circumcision**	**No**	229 (96)	122 (94)	351 (95)	0.30
	**Yes**	9 (4)	8 (6)	17 (5)	
**# of sexual partners/year**	**More than 1**	188 (80)	87 (68)	275 (76)	**0.01**
	**1 or less**	47 (20)	41 (32)	88 (24)	
**Condom use**	**Always**	75 (32)	38 (29)	113 (31)	0.54
	**Not always**	157 (68)	92 (71)	249 (69)	
**Penile discharge**	**Yes**	38 (16)	28 (22)	66 (18)	0.17
	**No**	200 (84)	101 (78)	301 (82)	
**Genital ulcers**	**Yes**	23 (10)	18 (14)	41 (11)	0.21
	**No**	215 (90)	111 (86)	326 (89)	
**HIV tested**	**Yes**	162 (68)	52 (40)	214 (58)	**<0.01**
	**No**	75 (32)	78 (60)	153 (42)	

**Table 2:** Perceived benefits of circumcision by national origin.

Category	Subcategory	Dominican	Haitian	Total	x^2^ (p value)
**MC improves hygiene**	Yes	175 (74)	89 (71)	264 (73)	0.43
	No	60 (26)	37 (29)	97 (27)	
**MC reduces STI**	Yes	64 (27)	47 (38)	111 (31)	**0.04**
	No	170 (73)	77 (62)	247 (69)	
**MC reduces HIV**	Yes	46 (20)	30 (24)	76 (21)	0.37
	No	187 (80)	96 (76)	283 (79)	
**MC reduces penile cancer**	Yes	71 (31)	41 (35)	112 (33)	0.43
	No	157 (69)	75 (65)	232 (67)	
**MC reduces pleasure**	Yes	109 (50)	49 (39)	158 (46)	**0.04**
	No	108 (50)	77 (61)	185 (54)	

Most men (73%) thought that MC improved hygiene (table 2) and approximately one third knew that it reduces the risk of penile cancer or STI. A small percentage (21%) knew that MC helps prevent HIV infection. Just under half (46%) reported that MC reduces sexual pleasure and 52% thought that women enjoy sex more with an uncircumcised man. A greater proportion of Dominicans than Haitians thought that MC decreases sexual pleasure (p = 0.04). More Haitians reported that the procedure decreases the risk of acquiring a STI (p = 0.04).

Self reported male-to-female anal intercourse was high (40%). Only three individuals identified themselves as “bisexuals” and none of the participants reported sexual intercourse exclusively with other men.

Both groups reported a high rate of inconsistent condom use with 68% of Dominicans and 72% of Haitians answering “never” or “sometimes” when asked about frequency of condom use. The self-reported history of penile discharge was 16% vs. 22% and of genital ulcerative disease was 10% vs. 14% in Dominicans and Haitians respectively.

### Willingness to be circumcised (table 3)

#### Before the information session

When first asked, 29% of participants reported that they would be willing to be circumcised if the procedure was offered to them. Overall, Haitian nationals (OR = 1.89, 95% CI 1.18–3.02) and younger men (OR = 1.65, 95% CI 1.02–2.66) were more agreeable to be circumcised when compared to their Dominican and older counterparts.

**Table 3:** Willingness to be circumcised by demographic characteristics and perceived benefits of the procedure (unadjusted OR).

		Yes	No	Total	OR	95% CI	P value
**Willing to have a circumcision, if offered (Before explaining the benefits)**		103 (29)	250 (71)	353			
**Willing to have a circumcision, if offered (After explaining the benefits)**		236 (67)	115 (33)	351			
**Nationality**	**Haitian**	47 (38)	77 (62)	124 (35)	**1.89**	**(1.18**–**3.02)**	**<0.01**
	**Dominican**	56 (24)	173 (76)	229 (65)			
**Age**	**Younger**	69 (33)	138 (67)	207 (59)	**1.65**	**(1.01**–**2.66)**	**0.04**
	**Older**	34 (23)	112 (77)	146 (41)			
**Education**	**<8^th^ grade**	48 (27)	129 (73)	177 (54)	0.80	(0.50–1.29)	0.36
	**>8^th^ grade**	48 (32)	103 (68)	151 (46)			
**Religion**	**Catholic**	44 (31)	97 (69)	141 (40)	1.17	(0.73–1.86)	0.51
	**Non-Catholic**	59 (28)	152 (72)	211 (60)			
**Marital status**	**Single**	58 (31)	130 (69)	188 (53)	1.19	(0.75–1.89)	0.46
	**Married**	45 (27)	120 (73)	165 (47)			
**MC improves hygiene**	**Yes**	88 (35)	161 (65)	249 (72)	**2.99**	**(1.63**–**5.49)**	**<0.01**
	**No**	15 (15)	82 (85)	97 (28)			
**MC reduces STI**	**Yes**	42 (41)	61 (59)	103 (30)	**2.16**	**(1.32**–**3.53)**	**<0.01**
	**No**	58 (24)	182 (76)	240 (70)			
**MC reduces HIV**	**Yes**	29 (42)	40 (58)	69 (20)	**2.08**	**(1.20**–**3.61)**	**<0.01**
	**No**	71 (26)	204 (59)	275 (80)			
**MC reduces penile cancer**	**Yes**	39 (38)	63 (62)	102 (31)	**1.98**	**(1.20**–**3.28)**	**<0.01**
	**No**	54 (24)	173 (76)	227 (69)			
**MC reduces pleasure**	**Yes**	31 (20)	122 (80)	153 (47)	**0.41**	**(0.25**–**0.67)**	**<0.01**
	**No**	67 (38)	108 (62)	175 (53)			

Men who ascribed a benefit to MC in terms of improving hygiene (OR = 2.99, 95% CI 1.63–5.49) or reducing the risk of acquiring a STI (OR = 2.16, 95% CI 1.32–3.53), HIV (OR = 2.08 95% CI 1.20–3.61) and penile cancer (OR = 1.98, 95% CI 1.20–3.28) favored the procedure. The level of acceptability was lower for those who reported that MC decreases sexual pleasure (OR = 0.41, 95% CI 0.25–0.67).

We examined the effect of sexual health and sexual behavior on the participant's willingness to be circumcised. There was no significant difference in acceptability between individuals who practiced anal sex (OR = 1.29, 95% CI 0.79–2.09) and those who did not. Similarly, a reported history of a STI (OR = 1.12, 95% CI 0.62–2.01) or penile discharge (OR = 1.27, 95% CI 0.71–2.27) was not associated with their willingness to be circumcised.

#### After the information session

The percentage of men willing to be circumcised increased to 67% after an information session describing the benefits of the procedure. The difference in acceptability between nationalities was nullified after this information was provided (OR = 1.02, 95%CI 0.64–1.62). The difference between younger and older men persisted (OR = 1.68, 95% CI 1.07–2.64). The educational level, religion and marital status of the participants were not associated with the acceptability of the procedure. 74% of participants reported that they would agree to circumcise their sons.

Stratifying by nationality and age group, prior to the information session, there were no differences between younger and older Haitians in willingness to be circumcised (OR = 1.01, 95% CI 0.47–2.14) but younger Dominicans were twice as likely to accept MC when compared to older Dominicans (OR = 2.17, 95% CI 1.14–4.12). Results were similar after the information session (younger Dominicans versus older OR = 2.33, 95% CI 1.32–4.09).

### Multivariate analysis

In the final adjusted multivariate model (table 4) the strongest predictors of men's acceptability of circumcision, before the information session, were Haitian nationality (OR = 1.86, 95% CI 1.01–3.41), thinking that circumcision improves hygiene (OR = 2.78, 95% CI 1.29–6.0) and not believing that having a circumcision decreases sexual pleasure (OR = 2.18, 95% CI 1.20–3.94). Education and age were not associated with circumcision preference. There was a tendency for those who knew that MC reduces the risk of STI and cancer to favor the procedure.

**Table 4:** Willingness to be circumcised. Multivariate analysis.

Variables		Adjusted OR (95% CI)	p-value
**Nationality**	**Haitian**	**1.86 (1.01–3.4)**	**0.05**
	**Dominican**		
**Age**	**Younger**	1.37 (0.40–1.32)	0.29
	**Older**		
**Education**	**>8th grade**	1.15 (0.64–2.07)	0.64
	**<8th grade**		
**MC improves hygiene**	**Yes**	**2.80 (1.29–6.0)**	**0.01**
	**No**		
**MC reduces STI**	**Yes**	1.62 (0.77–3.41)	0.21
	**No**		
**MC reduces HIV**	**Yes**	1.01 (0.43–2.39)	0.98
	**No**		
**MC reduces cancer**	**Yes**	1.44 (0.74–2.81)	0.28
	**No**		
**MC reduces pleasure**	**Yes**	**0.50 (0.25–0.83)**	**0.01**
	**No**		

## Discussion

In the present study, the proportion of men who were willing to be circumcised when first asked was 29%. This percentage is lower than in studies conducted in Africa where the median acceptability was about 65% (range 29–87%) [9]. Nevertheless, acceptability increased to 67% after participants were provided with information about the benefits of the procedure. Such increment was also observed in previous studies done in Botswana [18] and Thailand [15] where the willingness to be circumcised went from 61% to 81% and from 14% to 66%, respectively, after a similar information session.

The marked difference in acceptability before and after the information session suggests that lack of knowledge about the benefits of MC is an important factor in the initial response rates. However, other elements merit consideration. For instance, in some African countries, circumcision is widely practiced and is regarded as an important aspect of ethnic identity [9]. Over 30% of participants in several of the African acceptability studies were already circumcised at the time of the interview [19]–[20], in contrast to our study, where only 5% of men were circumcised. In communities with higher rates of MC, the uncircumcised man may be more aware of the procedure and is likely to have heard positive references from someone who is circumcised. MC in the Dominican Republic does not have a specific cultural significance and is not routinely performed, except when medically necessary. Circumcised males are rare exceptions and most people are even unaware that the procedure is performed for medical or cultural reasons. Naturally, men in these communities would be less likely to agree to a surgical procedure that is unknown to them.

Knowledge about the benefits of MC was low among study participants. Although the majority of individuals reported knowing that MC improves genital hygiene (73%), fewer than one third knew that it helps decrease the risk of penile cancer, STI and HIV. In the adjusted analysis, participants who reported that circumcision improves hygiene were almost three times more likely to accept the procedure and those who thought that it decreases pleasure were significantly less likely to agree to be circumcised.

The results of this study indicate that education can be an effective component of an intervention designed to increase the acceptability of MC among men in the DR. The question then becomes- who should be the target of such intervention? Before any information is provided, Haitians are more willing to be circumcised; however, Dominicans reach similar levels of acceptability when informed of the benefits. Although younger Dominicans appear more willing, the reluctance of older Dominicans may be contributing to the differences observed across age groups. The latter group is an important target because they are the parents of children and newborns who could benefit from the procedure. Even when older Dominicans are not willing to be circumcised themselves, they favor circumcising their children. Similar findings were reported in some of the African studies where adults were more agreeable to the procedure for their children rather than themselves [8].

This study has several strengths: men from a broad geographic area of the province were surveyed, we had high participation rates and the study allowed for a comparison between Dominicans and Haitians. The limitations are the inherent problems of the cross-sectional design; the pitfalls of using a convenience sample to extrapolate results to the general population, which may have led to selection bias favoring participants of a lower socioeconomic stratum; the fact that the information collected was based on reported behaviors and the limited availability of Creole translators, which made it harder to communicate with a very small number of Haitians who did not speak Spanish.

In summary, after considering the limitations of the study, we believe that an important proportion of sexually active men in the DR may be willing to be circumcised if educational resources detailing the benefits of the procedure are made available. In view of the high rates of inconsistent condom use, these educational activities or resources are an excellent opportunity to teach about sexual health and reinforce safe sex practices. Obviously, the feasibility of rolling out circumcision services in the DR would depend on a number of factors of which men's acceptability is only one. A feasibility study evaluating some of these factors was conducted by our team and has been reported elsewhere [21].
